# Exploring the Perceptions and Behaviours of UK Prescribers Concerning Novel Lipid-Lowering Agent Prescriptions: A Qualitative Study

**DOI:** 10.3390/pharmacy12040104

**Published:** 2024-07-03

**Authors:** Sarah Baig, Shahrauz Mughal, Yousuf Murad, Mandeep Virdee, Zahraa Jalal

**Affiliations:** 1 School of Pharmacy, Institute of Clinical Sciences, College of Medical and Dental Sciences, Birmingham B15 2TT, UKz.jalal@bham.ac.uk (Z.J.); 2 Worcestershire Acute Hospital Trust Woodrow Drive, Redditch B98 7UB, UK; yousuf.murad@nhs.net; 3 Royal Wolverhampton NHS Trust, Wolverhampton WV10 0QP, UK; m.virdee@nhs.net

**Keywords:** lipid-lowering therapy, competence, shared decision making, education, training

## Abstract

Reducing low-density lipoprotein cholesterol levels lowers the risk of atherosclerotic cardiovascular disease. With the current and future portfolios of emerging lipid-lowering therapies included in various national and international guidelines, the objectives of this study were (i) to investigate the perceptions of UK prescribers’, including doctors, pharmacists, and nurses, on current lipid management for cardiovascular diseases and prescriptions of novel lipid-lowering therapies, and (ii) to explore the challenges and facilitating factors of prescribing novel lipid-lowering therapies through qualitative interviews. Qualitative semi-structured interviews with twelve medical and non-medical prescribers were conducted, around 20–30 min in length. The interviews were audio-recorded and transcribed on an online platform. A thematic analysis was deployed. Four major themes emerged from the analysis: (1) prescribing barriers; (2) prescribing enablers; (3) inter-profession variability; and (4) health literacy. These themes highlighted the contrast between the need for optimal shared decision making and the various constraints in practice. Participants expressed their inexperience with novel lipid-lowering therapies and acknowledged the requirement and importance of these agents for primary cardiovascular disease prevention. Participants recognised confidence and competence as key drivers for prescribing therapies and welcomed further education and training to enhance their skillset. Patients’ misconceptions towards current lipid-lowering therapies contributed to their refusal of newer agents, highlighting a requirement to improve patient education. Targeting communities through awareness campaigns was identified as a viable solution.

## 1. Introduction

Cardiovascular disease (CVD) remains the leading underlying cause of mortality worldwide [1]. Dyslipidaemia, particularly low-density lipoprotein cholesterol (LDL-C) and non-high-density lipoprotein (non-HDL) are established as major modifiable risk factors for the primary and secondary prevention of atherosclerotic cardiovascular disease (ASCVD) [1,2]. Reducing non-HDL remains a vital therapeutic target for patients with existing risk or those who are at risk of developing ASCVD, as it pertains to lower cardiovascular events (CVEs) [2]. For patients who struggle with conventional lipid-lowering therapies (LLTs) or who are at an extremely high risk of experiencing CVEs, further LLTs are required to achieve a greater absolute-risk reduction.

Initial statin monotherapy has long been established as a first-line treatment in lipid modification causing a decrease in LDL-C in the range of 30–63%, depending on the intensity [3,4]. Statins competitively target the reduction in LDL-C by inhibiting 3-hydroxy-3-methylglutaryl coenzyme A reductase (HMG-CoA reductase), a catalytic enzyme involved in cholesterol biosynthesis. This inhibition ultimately causes a decrease in cholesterol synthesis in the liver and a greater clearance of LDL-C from the bloodstream [4].

Additional statin benefits include a reduction in total cholesterol and very-low-density lipoprotein and triglycerides, whilst increasing HDL concentrations [5]. Ezetimibe inhibits intestinal cholesterol absorption and is a second-line treatment that can be used as a monotherapy or in conjunction with statins amplifying LDL-C reduction by approximately 14 percent [4]. Alirocumab and Evolocumab are proprotein convertase subtilisin kexin type 9 (PCSK9)-targeting monoclonal antibodies, which prevent PCSK9 complexing with LDL receptors. This complex initiates the degradation of the receptor, decreasing the total available receptors capable of clearing LDL-C from the plasma [6].

Although statin monotherapy is the most widely utilised lipid therapy as per the national guidance, there are many circumstances resulting in sub-optimal dosing or the discontinuation of therapy. Poor adherence and intolerance to statins can often be attributed to musculoskeletal side-effects ranging from myalgia to rhabdomyolysis, which feature in >90% of statin-discontinuation cases [7]. Additionally, the guidelines from the European Society of Cardiology justify the introduction of high-intensity statins to attain a >50% LDL-C reduction to further reduce the risk of ASCVD and improve patient outcomes [8]. However, sub-optimal treatment creates an obstacle for clinicians in lipid management and the overall risk reduction in CVEs, leading to a disparity between LDL-C reduction in practice compared to treatment objectives [9]. Likewise, an average LDL-C of 3.0–3.5 mmol/L before treatment requires targets as low as 1.4 mmol/L, which are not achievable with monotherapy [10].

Combination therapies have been recommended by various international and national guidelines to enhance therapeutic benefits, however this approach is underutilised for reasons including cost, pharmacovigilance, and poor health literacy [11,12,13]. Nonetheless, prescribing triple therapy, including a high-intensity statin, ezetimibe, and PCSK9 inhibitors, concomitantly does not confer a significant reduction in LDL-C in certain patient groups, resulting in the pursuit of novel LLTs [14].

Novel LLTs have introduced a multimodal approach of targeting ASCVDs, leading to the introduction of numerous original and upcoming therapies, which are expressed in Appendix A and Appendix B. Although recent research has explored current and emerging novel LLTs alongside patient perceptions, there has been limited research investigating the perceptions of prescribers of novel agents. This may be due to their more recent integration into UK practice [15,16,17,18].

Inclisiran is an injectable, small, interfering ribonucleic acid treatment exerting its mechanism of action intracellularly by selectively targeting PCSK9 messenger ribonucleic acid. This ultimately inhibits PCSK9 protein translation, resulting in the upregulation of hepatocyte LDL receptors enhancing LDL plasma clearance [19,20]. Similarly, bempedoic acid is an orally administered prodrug inhibiting adenosine triphosphate citrate lyase. Its activation in the liver ultimately improves LDL plasma clearance through the upregulation of LDL receptors. Its specific liver activation as opposed to muscle cells has been envisioned as a potential advantage in patients with statin-associated myalgia [19,20]. Evinacumab is a monoclonal antibody approved by the European Medicines Agency and the United States Food and Drug Administration. The National Institute for Health and Care Excellence (NICE) guidelines will soon be updated to include Evinacumab in 2024 [19,20,21]. This medication works by targeting free angiopoietin-related protein 3 (ANGPTL3) and sterically hindering the inhibition of ANGPTL3-induced lipases, resulting in an increase in lipase activity. This increases the hydrolysation of triglycerides, which reduces circulating LDL-C independently of the number of available LDL receptors capable of removing LDL-C from the bloodstream [19,20]. Omega-3 fatty acids, like icosapent ethyl, have demonstrated a reduction in plasma triglyceride levels and triglyceride-rich lipoproteins, thus delaying atherosclerosis [20].

A caveat with novel lipid therapy prescriptions is the lack of adverse drug reactions (ADRs) reported in the wider population from real-world data. Idiosyncratic ADRs cannot be predicted in relation to pharmacological responses resulting in a decrease in prescribing activity [22].

In the UK, the spontaneous reporting of ADRs can be completed through the Yellow Card Scheme governed by the Medicines and Healthcare Products Regulatory Agency and the Commission on Human Medicines. Although pharmacovigilance remains a significant responsibility of all healthcare professionals (HCPs) and patients, ADRs are underreported, potentially impacting novel therapy prescriptions [22].

Non-medical prescribing (NMP) has rapidly evolved since its inception in the UK in 1992 [23]. Initially, doctors or dentists were the only HCPs authorised to prescribe therapies. However, pharmacists, nurses, and selected allied health professionals are now capable of prescribing drug therapies [23,24,25]. After September 2026, newly qualified pharmacists in the UK will be prescribing independently from the day of their registration [26]. Embedding independent prescribing pharmacists and nurses across primary and secondary care sectors increases patients’ access to healthcare [25,26]. With this seismic shift towards prescribing across multiple healthcare professions, it is imperative to investigate the perceptions of prescribing novel LLTs.

Primary care services in the UK, are incentivized nationally by the Quality and Outcomes Framework (QOF) to ensure high-quality care. QOF consists of multiple target indicators and domains to intervene in various long-term-condition management effectively to improve the overall quality of care [27]. Cholesterol and lipid management is one of the clinical domains expressed by the QOF. Novel LLTs may have a place in therapy, where statins are contraindicated or not tolerated in line with the NICE guidelines [27]. Two new cholesterol indicators have been added to the QOF, with the changes in the General Practice contracts in 2023/2024 potentially leading to more effective lipid optimisation and prescriptions of novel LLTs [27,28]. In anticipation of these modifications, this study will explore UK prescribers’ current views of prescribing novel LLTs in order to address the research gap in the wider literature and highlight the current experiences of UK prescribers in relation to novel LLTs, which may form a basis to improve prescribing practice.

### Aims and Objectives

1—To investigate perceptions of UK prescribers’, including doctors, pharmacists, and nurses, of current lipid management for cardiovascular diseases and prescribing novel lipid-lowering therapies.

2—To explore the challenges and facilitators of prescribing novel lipid-lowering therapies through qualitative interviews (see Appendix A, Appendix B and Appendix C).

## 2. Materials and Methods

A qualitative study was conducted in the UK, West Midlands, utilising semi-structured interviews to evaluate prescribers’ perspectives regarding novel LLTs in relation to dyslipidaemia and CVE reduction. Various prescribers from all sectors were contacted by email or word-of-mouth through convenience sampling to participate in the study. Demographics and responses were gathered from eight questions focusing on two major concepts: challenges and facilitators of prescribing current and novel LLTs. The interview questions were piloted with two pharmacists to ensure coherence and the appropriateness of the discussion points prior to data collection between the beginning of October 2023 and the end of November 2023. The results obtained from the pilot interviews are excluded from this study. A thematic analysis was performed on the interview transcripts to identify common themes and patterns to synthesise real-world experiences with prescribing novel LLTs.

Ethical approval: The ethics sub-committee of the University of Birmingham School of Pharmacy granted ethical approval for the study (ethics no. 23/24-010).

### 2.1. Procedure

Prescribers were invited via email or word of mouth. The HCPs consenting to take part were briefed on the study, and a participant information sheet was provided alongside a consent form either as a virtual or hard copy based on their preference. Interviews were scheduled for a convenient time for each participant. After verbal and written consent were determined, semi-structured interviews were undertaken, lasting around 20–30 min. Data were obtained between October and November 2023. The interviews were conducted via an online Zoom platform by researcher SM. Gratitude was expressed to all participants for taking part in the study. The research regarding real-world experiences of prescribers towards novel LLTs is limited, therefore an inductive research approach was adopted (see Supplementary Material).

### 2.2. Data Analysis

Thematic analysis of interview transcripts. All interviews were audio, video-recorded, and transcribed via an online Zoom platform, and thematic analysis was performed manually using the principles of Braun and Clarke (2008) [29]. All transcripts were reviewed on three occasions alongside the audio recording for familiarity by researcher SM to ensure the accuracy of the transcripts prior to coding themes. Upon establishing familiarity with all transcripts, the identification and coding of themes and subthemes were determined. Initial themes were identified from individual transcripts with similar codes grouped together to ease the comparison. All established themes were assessed and refined to ensure consistency and relevance.

Data reporting was performed to act in accordance with the Consolidated Criteria for Reporting Qualitative Research guidelines and checklist [30].

## 3. Results

Twelve prescribers participated in the interviews. All participants were currently based in primary care, however experience in secondary care was exhibited by all interviewees. Participant demographics are highlighted in Table 1.

### 3.1. Participants

**Figure 1:**
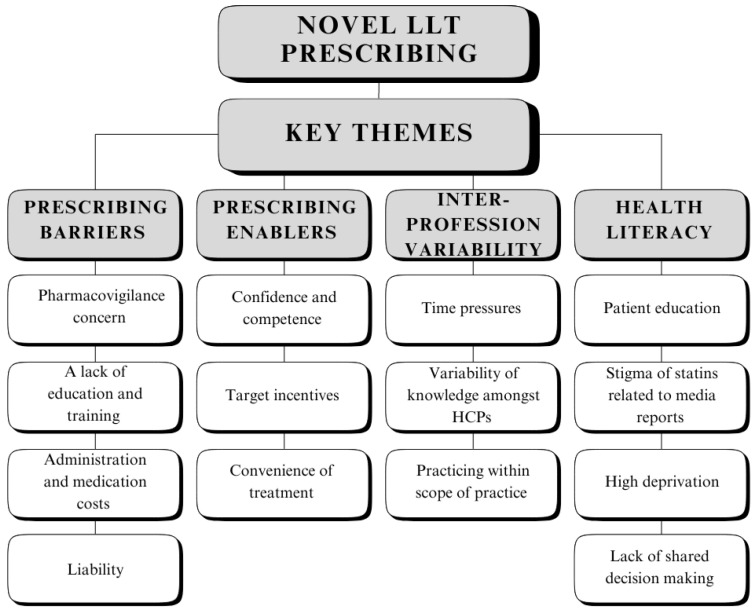
Flowchart demonstrating theme and subtheme identification.

A relatively small sample (n = 12; 7 females, 5 males) was employed consisting of UK general practitioners (GPs), pharmacists, and a nurse. Inclusion criteria included: practicing HCPs, completion of a prescribing qualification, and current engagement in primary or secondary care located in the West Midlands region. Community pharmacists, who are not involved in managing patients were excluded from the study. All HCPs were recruited through convenience sampling by email and word of mouth (refer to Figure 1 and Table 1).

### 3.2. Themes

Four major themes materialised from the thematic analysis of the interview transcripts: 1. prescribing barriers; 2. prescribing enablers; 3. inter-profession variability; and 4. health literacy (refer to Table 2).

#### 3.2.1. Theme 1—Prescribing Barriers

Primary care prescribers displayed hesitancy in prescribing novel LLTs, in particular, inclisiran more so than bempedoic acid, whilst PCKS9 inhibitors were found to be primarily initiated in secondary care. Prescribers reported their inexperience with novel LLTs, for example bempedoic acid, which was prescribed only a “handful” of times by some participants. The reluctancy to prescribe novel LLTs, especially inclisiran, stemmed from the black triangle status and lack of safety data regarding use in the wider population, resulting in a “cautious” approach:

“Generally, as a profession, pharmacists are quite risk averse. So, they are quite reluctant to prescribe the newer agents. It could be because of pharmacovigilance… obviously they’re very new, they are black triangle drugs. Therefore, that could be a deterrent and may also almost put people off from prescribing them and not just prescribers, but also patients because there’s not much experience with using these agents and patients might be reluctant to try them as well.”

(Participant 1)

Participants mentioned training was received for novel LLTs by the drug company’s representatives alongside online workshops. However, the need for additional education and training to increase confidence and competence was illustrated by all participants, especially for up-and-coming prescribers and some practice sites:

“…It’s very early days. It’s a black triangle drug [inclisiran]. A lot of practices are cautious about its use, even though the initial data shows it’s very effective. There’s not much safety data around it. So hence there’s not much prescribing, but I think it’s developing, and I suppose if there is more exposure, more sort of you know, training or shadowing in prescribing, I don’t see there’d be a problem. But a lot of practices don’t want to initiate because they’re not happy to follow up, or for the GPs to prescribe.”

(Participant 6)

A solution to prescribers’ inexperience with novel LLTs was utilising the knowledge of senior team members. This approach had varying views across the interviews:

“…There’ll always be somebody who’s got experience. So, if you need to pick someones’ brains, or go and shadow somebody to learn about it yourself, you can.”

(Participant 8)

“A GP sent through a query to say, oh, is this [bempedoic acid] hospital only? so I think the fact that it’s quite new, the fact that it’s not used much. GPs aren’t aware of it. They don’t know what it is. Is it hospital only or is it specialist only?”

(Participant 6)

Numerous factors were considered by all participants, with cost established as a major concern: “It’s quite costly, whereas statins are relatively lower in cost, costly in terms of the drug, costly in terms of, you know, administrating costs as well. You need nurses to do it, or the GP”.

(Participant 9)

Liability was a factor overshadowing prescribers’ thoughts, which was highlighted by Participant 6:

“If there’s a GP that’s prescribing it going forward. If something was to go inadvertently wrong. He or she would have to, then you know, justify that. So, for that reason they’re not, they’re not not all that keen.”

Overall, a lack of training and experience resulted in a reduction in confidence and competence to prescribe novel LLTs. The concept of pharmacovigilance was considered alongside potential litigation in any unfortunate event.

#### 3.2.2. Theme 2—Prescribing Enablers

This theme highlighted factors aiding participants’ prescribing decisions. Decision making was heavily influenced by the education and training received, which facilitated prescribers’ willingness to prescribe novel LLTs where appropriate: “I think bempedoic acid is straightforward. It’s just about education. It’s just about information sessions which we’re doing currently in practices” (Participant 6). Prescribers recognised working within their scope of practice was key to improving patient outcomes, and experience played a crucial role:

“Having the experience allows you to have that confidence because you’ve either seen it before and you know how to deal with it. You feel more confident, you trust your decisions more.”

(Participant 1)

Although novel LLTs have been recommended by NICE guidance, the two additional 2023/2024 QOF indicators have assisted prescribing novel LLTs, especially in cases where aggressive lipid reduction is required. Participant 12 reported:

“QOF now has indicators for cholesterol management and also there’s a local prescribing incentive scheme where for existing patients with cardiovascular disease, we need to try and improve the lipid management, particularly non-HDL.”

Prescribers heavily involved in lipid reviews and management because of the QOF had a better understanding and gauge of their confidence in prescribing novel LLTs.

The safe and appropriate prescribing of novel LLTs was established to be appropriate from a prescriber and patient perspective addressing concerns around polypharmacy and high lipid levels. The option to prescribe novel LLTs for patients’ convenience to increase adherence was extremely valuable, “especially for patients who are either treatment resistant or have a high tablet burden” (Participant 1).

Although cost was perceived as a significant barrier, some prescribers viewed cost as a major facilitator:

“Ultimately, the cost of some of these medicines [novel lipid-lowering therapies], if they reduce a cardiac event, that would be a lot more cost-efficient.”

(Participant 10)

Overall, novel LLTs provided a possible treatment option for prescribers, especially for statin-intolerant patients and those who did not achieve the desired lipid target. The utilisation of the wider workforce as part of local and national incentive schemes grants further opportunities for prescribers to increase their competence with novel LLT prescribing.

#### 3.2.3. Theme 3—Inter-Profession Variability

The third theme illustrated various pressures and difficulties amongst participants, which directly influenced their ability to prescribe novel LLTs. The key pressure of time was recognised by most participants, which hindered the opportunities to potentially discuss novel LLTs, where they were appropriate. However, pharmacists were found to experience this difficulty far less than other professions:

“It could be that they [practice co-workers] don’t have time to review patients’ therapy because they’re too busy doing the routine work like the tasks and the Docman and various other things within the surgeries… As a pharmacist, I’m quite lucky cause I have a little bit more time than perhaps a medic would or a nurse, so I do get a bit of extra time to be able to engage in conversation with a patient.”

(Participant 1)

Time constraints resulted in reduced opportunities to engage with patients, where effective shared decision-making goals could not be agreed upon:

“… Patient education, unfortunately, that they don’t have it just due to time restraints”.

(Participant 5)

Novel LLTs were noted to be initially prescribed in secondary care and followed up in primary care, which a proportion of the participants were adjusting to; Participant 8 reported:

“… But we’ve seen it come down from again like the secondary care initiation from consultants. So, it’s something, I think we as like prescribers in primary care, so like doctors, pharmacists, advanced practitioners, It’s something we don’t tend to prescribe, because that hasn’t, we were advised that this comes through secondary care.”

NMPs exercised additional caution when prescribing, which was further potentiated by novel LLTs:

“…We’ve done a six month prescribing qualification. But you know you do not go from, you know, from a six month prescribing qualification to being fully competent prescribing all drugs. So its a case of building your competence, building skills working on your scope of practice on a routine basis.”

(Participant 7)

All participants were conscious of the prescribing guidelines and the accelerated access collaborative pathway, but adjusted their prescribing behaviour if they operated in multiple practices. Not all practice sites were willing to prescribe novel LLTs in line with the national incentive scheme, whilst some practices exercised greater caution, where novel LLTs were discussed in a multidisciplinary team (MDT) fashion prior to initiation. For example, Participant 11 stated:

“We do that [initiation of novel lipid-lowering therapies] through an MDT. So we’d sit with the the GP and we sit with, you know… we have we’ve tried all these options. This is our next line of treatment. What are your thoughts here? It’s a green drug on our formulary, we can initiate it. Sometimes on those we write to our lipid clinic to get advice and guidance and then we start to initiate. So i’ve only done 2 of those [inclisiran]. But it’s been in conjunction, almost like a supplementary plan, like a care plan with a GP.”

To increase exposure to novel LLTs and overcome various prescribing barriers, shadowing members of the wider workforce was proposed as a solution alongside continuous professional development (CPD).

Participant 10 illustrated:

“I think there’s quite a lot of educational sessions, CPD sessions on managing high cholesterol. I think what would work slightly better is especially with allied health care staff is that if they work alongside a GP when they’re managing cholesterol, because I think what I tend to find is that sometimes we need to adopt a more pragmatic approach.”

CPD was identified by participants to enhance personal growth; however, experienced participants identified the various drawbacks associated with CPD as a professional development tool:

“Competence is self-defined when you become a qualified pharmacist, and that that almost creates a barrier because you’re relying on that person to either A, upskill themselves through CPD or B, ask for that for that training and development which can sometimes be quite difficult for some people to access as well due to time and other other reasons.”

(Participant 1)

Overall, certain HCPs faced different challenges in comparison to their colleagues, especially NMPs who, due to a lack of initial prescribing experience, exhibited greater caution. To upskill themselves, working in collaboration with other MDT members and using the wider workforce were described as viable solutions. Senior members within practices themselves also required further training and experience with novel LLTs, which can be attributed to their recent transfer from the secondary care sector.

#### 3.2.4. Theme 4—Health Literacy

The final theme represented patients’ roles in novel LLTs and its effects on prescribers. A lack of patient awareness and education regarding cholesterol management was recognised by all prescribers, where patients were quick to decline contemporary LLTs alongside novel treatment. This mostly occurred in the initial stages of treatment or a “few months later”, as patients stated they “don’t need it now.” Participant 9 reported:

“They [patients] don’t think it’s relevant like, if you know when you can’t see something, you’re less likely to take it. If you can see that actually, you’ve got a rash, you’re more likely to use a cream. Whereas sometimes I think with these underlying conditions like blood pressure, lipids etc… because they can’t really see those symptoms, to them they feel fine. So that affects adherence.”

Stigma within the media regarding statin treatment was established as a core belief amongst patients refusing contemporary and novel LLTs. Participants had to debunk “myths” associated with statins and address patient concerns to support reaching a shared decision. This problem was prevalent, particularly amongst elderly patients. Statin misconceptions resulted in patients’ refusal of all LLTs or as motivators for novel treatment:

“So with patients, it really varies. Some patients are quite eager. So they read about a lot. There is that myths about statins, and they do read up quite a lot about the newer therapies. Some of them are quite keen to have like an injection, and things like that”

(Participant 8)

Patients from affluent areas were found by participants to be well informed regarding novel agents and less likely to believe misinformation, whereas patients from deprived areas struggled to reach shared decisions. Patients from deprived areas allowed HCPs to make clinical decisions on their behalf, whilst informed patients questioned the participants to improve their understanding. For all patients to have equal access to treatment, various solutions were recommended by the participants, such as increasing awareness in the general population and addressing patient concerns to ultimately improve shared decisions and adherence:

“…So as part of like effective prescribing. We need to make sure that our patients are empowered about the decisions that they make. The way we can do that is, by engaging with some of these communities, whether that’s, you know, communicating things like the newer agents in different languages, using interpreters or helping to educate them around lipid management that that just doesn’t just include the drugs, but also the lifestyle.”

(Participant 1)

“I think it’s about having that discussion with a patient early on and explaining and sharing your concerns with them. Because if you do that, you know they’ll understand what you’re worried about because they don’t understand what total cholesterol of six is. They don’t understand what HDL is. They don’t understand the significance of that LDL but if you sit there, spend a bit of time with them initially and explain that to them… if you feed their mind with the right information, they’ll go away thinking about it, and they’ll come back more likely to accept treatment and more likely to be concordant with treatment.”

(Participant 10)

In summary, a portion of patients werequick to dismiss statins alongside potential novel LLT initiation due to misconceptions relating to “aches and pains”. Medicines equity for all patients were established as a core belief for all participants, especially for patients living in areas of high deprivation or low Index of Multiple Deprivation (IMD), where shared decision making was difficult to establish. The importance of increasing the awareness of cholesterol management in more deprived communities was emphasised as a potential solution.

## 4. Discussion

There has been extensive research exploring the efficacy of novel and upcoming LLTs. Other research has investigated patient perceptions of novel agents. Within patient perception research, this has primarily focused on injectable lipid novel therapies [15,16,17,18]. To the best of our knowledge, this is one of the first qualitative studies exploring UK prescriber perceptions of all available novel lipid agents. The following discussion will examine the four identified major themes: 1. prescribing barriers; 2. prescribing enablers; 3. inter-profession variability; and 4. health literacy. This study will conclude with a Section 4.5 alongside the implications for future practice and research.

### 4.1. Prescribing Barriers

Prescribers in this study stated that safety profiles and their personal experience with specific agents heavily affected their prescribing decisions. A lack of education and training with novel LLTs influenced prescribers’ decision making, which has been highlighted in previous studies, where prescribers have a preference or habit to prescribe certainmedications which they are comfortable prescribing which are clinically effective and safe drug therapies [31]. The importance of pharmacovigilance and error reporting with newer agents was considered by prescribers, where the underreporting of potential side-effects was a significant concern, especially amongst certain patient groups. A potential solution included educating patients on the significance of ADR self-reporting systems, which is in line with a systematic review of 26 studies that found patient knowledge to be an impactful factor [32]. A previous qualitative study highlighted GPs’ and primary care organisations’ perspectives on cost, which displayed greater leniency than what was found in this study [33]. Displaying excessive caution when prescribing newer agents and frequently prescribing conventional therapy may overlook therapeutic opportunities; therefore, an informed decision is advantageous [34]. A limitation of this study includes a lack of professional diversity in the prescribers and an abundance of pharmacists. Pharmacists play a critical role in cost-effective prescribing as part of medicine optimisation, which may align with cost appearing as a recurrent subtheme [35,36].

### 4.2. Prescribing Enablers

In the current study, prescribers alluded to factors that eased their transition into prescribing novel LLTs. Initially, novel LLTs were prescribed in secondary care by consultants prior to continuation in primary care. However, with the recent implementation of novel LLTs into lipid management in primary care, prescribers have been required to rapidly upskill [37]. Prescribers reported additional training through in-person and online teaching events to increase their competence, which, in turn, enhanced their confidence to prescribe novel LLTs. Although prescribers’ experiences with novel LLTs began to grow, their training needs were not fully met. This is supported by a scoping review that highlighted the importance of self-awareness to support prescribing decisions, stating competent pharmacists often state they require additional training [38].

Distinctive views were expressed by the interviewees regarding incentive arrangements, particularly the QOF and local incentive schemes. Prescribers reported the GP contractual changes regarding cholesterol management alongside the incentivisation of inclisiran may aid in prescribing novel LLTs, especially in cases where aggressive lipid reduction is required. Overprescribing due to incentive targets was highlighted as a potential concern from the participants; however, this link remains uncertain in the wider literature and requires further research [39]. Patient’ convenience was considered a crucial consideration in prescribing novel LLTs where appropriate; previous studies highlighted the challenges associated with polypharmacy and pill burden and the importance of deprescribing, which novel LLTs address [40].

### 4.3. Inter-Profession Variability

Different prescribing professions illustrated various prescribing pressures. Prescribers reported time as a considerable challenge due to the difficulty of articulating the necessary information to reach a shared decision within the consultation timeframe. Some participants relayed information on behalf of their colleagues to support this statement, which may reduce the reliability of the findings. However, these discoveries are supported in the wider literature [41]. NMPs acknowledged their longer consultation times to discuss novel LLTs, which was perceived positively by patients. Previous evidence highlighted that extended consultation times with NMPs were recognised positively by patients, which is in line with this study’s findings [42,43]. However, a study by Gerard et al. discovered that consultation times had no overall influence on patient satisfaction [44].

Participants acknowledged differences in expertise between healthcare professions and the concept of practicing within their personal scope of clinical competence as a crucial safety element, regardless of profession. Although independent prescribing enabled NMPs to practice autonomously, greater confidence was reported within their prescribing area of competence, whilst significant caution or refusal was expressed outside their clinical area. This included prescribing novel LLTs. These findings are supported by the Royal Pharmaceutical Society’s “competency framework for all prescribers” and a systematic review of 42 studies, which signified the importance of practicing within your “area of competence” [45,46]. Practicing within the framework elicits safe and effective prescribing, which is critical for novel LLTs due to their black triangle status and sparcity of safety data.

### 4.4. Health Literacy

All prescribers agreed on the importance of enhancing patient awareness and education, especially regarding debunking the misconceptions associated with LLTs to ultimately improve patients’ health outcomes. Previous studies illustrated low health literacy corresponded to inadequate self-health management and healthcare utilisation justifying participants’ reluctancy to prescribe novel LLTs, especially for patients from highly deprived areas [47]. Participants reported that some patients either outright refused conventional and novel treatments or, after an initial introduction to statins, discontinued treatment due to the side-effects associated with negative perceptions without consulting HCPs. These findings were supported by a 2020 study, where >90% of symptoms associated with statins were present, regardless of whether the patient took atorvastatin or a placebo, which was explained by the “nocebo effect” resulting in the discontinuation of treatment without seeking professional advice [48].

Patients residing in Lower layer Super Output Areas (LSOAs) in deprived areas across England encountered several healthcare inequalities, including healthcare experiences and disease outcomes, which participants understood and recognised as a concern [49]. These disparities nationwide spurred the development of the Core20PLUS5 initiative which aims to reduce healthcare associated inequalities. Several areas within the West Midlands are categorised within the Core20PLUS5 scheme, in which all study participants currently practice [50]. Many patients from these areas did not display a significant understanding of healthcare management and preferred the clinician’s direction in treatment. The lack of shared decision making and engagement with informed decisions from patients resulted in participants’ judgement to remain cautious when prescribing novel LLTs. A solution proposed by participants was to increase consultation time with patients, which is supported by the wider literature or through the use of patient awareness campaigns [47]. A potential solution wich can be proposed from an organisational point of view is the National Health Service Workforce Race Equality Standard data, which aims to incorporate additional employees from ethnic minorities [51]. This may aid in addressing language difficulties in order to establish improved communication with patients from diverse backgrounds to aid in informed decisions and novel LLT prescribing [47].

### 4.5. Strengths and Limitations

The key purpose of this study was to identify and explore prescribers’ in-depth real-world experiences of novel LLTs. Online interviews were conducted to achieve this after obtaining participants from convenience sampling. The possibilities of social desirability and recall bias cannot be excluded alongside researcher bias due to a single researcher conducting the interviews. Nonetheless, prescribers were based across multiple practices in the West Midlands, UK, with varied experiences and breadth of opinions potentially enhancing the generalisability of the findings. Researchers were conscious of reflexivity to minimise researcher bias throughout the interview and analysis process. Although, this study’s sample size is small, it is comparable to previous qualitative studies, which was appropriate to attain data saturation. Achieving saturation could possibly be due to most of the sample consisting of pharmacists [52,53]. Availability of secondary care prescribers and doctors limited their participation in this study. Therefore, these findings may not be applicable outside the West Midlands or in secondary care, which depicts the prescribing behaviour illustrated by the national novel LLT prescribing data [54,55,56].

### 4.6. Implications for Future Practice and Research

The findings of this study indicate a requirement for primary care prescribers to further develop their competence with novel LLTsto be able to provide necessary treatment where appropriate. This includes experienced prescribers who may benefit from an MDT model or peer-to-peer learning. Newly qualified prescribers may benefit from mentorship or through the identification of learning needs through CPD. Current misconceptions of contemporary treatments and subsequent perceptions of novel LLTs must be addressed to improve patient outcomes, which NMPs can use to their advantage due to their extended consultation timings. Adopting novel LLT prescribing as part of a wider team population focussed interventions may increase LLT prescribing rates where this is safe and appropriate, which may increase rates of novel LLT prescribing. Targeted patient education through outreach or patient awareness campaigns, especially in communities with high deprivation, may provide substantial benefits to both current and future treatment alongside encouraging self-management. Additional research is required in primary care with a diverse HCP sample to confirm this study’s findings. Similarly, research into secondary care HCPs and patient perceptions is necessary to increase the generalisability and validity of this study.

## 5. Conclusions

This study highlights primary care prescribers’ requirements for the additional development of novel LLT prescribing to enhance their confidence and competence. Time restraints along with a lack of robust safety data were some of the many challenges highlighted by prescribers, which influenced clinicians’’ willingness to prescribe novel lipid agents. Currently, prescribing novel LLTs where appropriate through a multidisciplinary team who with targeted work focussed on lipid lowering agents may reduce prescribing barriers. Improving patients’ views on concurrent statin therapy and overall self-management may provide substantial benefits for outcmes associated with LLTs. Further research into primary and secondary care alongside patient perceptions are required to establish if this current study’s findings are credible. In Summary, novel agents targeting dyslipidaemia are a rapidly developing field, and overcoming barriers for prescribing these agents, where possible, may lead to the improved patient access to these agents in practice.

## Figures and Tables

**Table 1:** Participant demographics.

Participant ID	Years of Practice	Years of Prescribing	Sex	Sector	Profession *	How Often Lipid-Lowering Therapy Is Prescribed
1	(15–20)	13	F	PC	Clinical pharmacist	Weekly
2	(0–5)	<1	F	PC	IP clinical pharmacist	Weekly
3	(30–35)	15	F	PC	IP nurse	Monthly
4	(0–5)	5	F	PC	Dr, GP registrar	Weekly
5	(0–5)	<1	F	PC	IP clinical pharmacist	Daily
6	(15–20)	10	M	PC	IP clinical pharmacist (clinical lipid ambassador)	Daily
7	(10–15)	5	M	PC	IP pharmacist	Weekly
8	(15–20)	7	F	PC	PCN lead clinical pharmacist	Weekly
9	(15–20)	0.25	F	PC	IP clinical pharmacist	Weekly
10	(15–20)	16	M	PC	Dr, GP	Daily
11	(20–25)	16	M	PC	Clinical pharmacist (pharmaceutical advisor)	Daily
12	(25–30)	16	M	PC	Clinical pharmacist	Daily

* Dr, doctor; GP, general practitioner; IP, independent prescribing; PC, primary care; PCN, primary care network.

**Table 2:** Theme and subtheme identification.

Major Theme	Subthemes
1. Prescribing Barriers	Pharmacovigilance concern A lack of education or training Administration and medication costs Liability
2. Prescribing Enablers	Confidence and competence Target incentives Convenience of treatment
3. Inter-profession Variability	Time pressures Variability in knowledge amongst HCPs Practicing within scope of practice
4. Health Literacy	Patient education Stigma of statins related to media reports High deprivation Lack of shared decision making

## Data Availability

The data presented in this study are available in the article.

## Supplementary Materials

The following supporting information can be downloaded at: https://www.mdpi.com/article/10.3390/pharmacy12040104/s1, Interview topic guide.

## Appendix A

**Table A1:** Novel lipid-lowering therapies in development stages III–IV.

Novel Therapy	Dose	Route	Drug Nature	Drug Target	Development Stage
Bempedoic acid	180 mg OD [57]	PO [57]	Small molecule [57,58]	ACLY [57,58]	III–IV [57]
CSL-112	6 g once weekly [57]	IV [57]	Plasma-derived apoA-I [57,58]	ApoA-1 [57,58]	III [57]
Evinacumab	15 mg/kg once monthly or 300 mg once weekly [57]	Sc [57]	Mab [57,58]	ANGPTL3 [57,58]	III–IV [57]
Icosapent ethyl	2 g BD [57]	PO [57]	Omega-3 fatty acid [57]	Not known [57,58]	III–IV [57]
Inclisiran	300 mg biannually [57]	Sc [57]	siRNA [57,58]	PCSK9 [57,58]	III–IV [57]
Omega-3 fatty acids (DHA and EPA)	4 g OD [57]	PO [57,59]	Omega-3 fatty acid [57,59]	Not known [57]	III–IV [59]
Olezarsen	50 mg monthly [57]	Sc [57]	ASO [57]	APOC3 [57]	III [60]
Pelacarsen	20 mg once weekly or 80 mg once monthly [57]	Sc [57]	ASO [57]	mRNA of LPA gene [57]	III [57]
Pemafibrate	0.4 mg (maximum daily) [61]	PO [58,61]	Fibrate [58]	PPARα [58]	III [58,61]
Volanesorsen	285 mg weekly or every 2 weeks [57]	Sc [57]	ASO [57,58]	APOC3 [57,58]	III–IV [57]

ACLY, adenosine triphosphate citrate lyase; ANGPTL3, angiopoietin-related protein 3; ApoA-I, apolipoprotein A-I; APOC3, apolipoprotein C3; ASO, antisense oligonucleotide; BD, twice daily; IV, intravenous administration; LPA, lipoprotein (a); OD, once daily; PO, oral administration; PPARα, peroxisome proliferator-activated receptor alpha; mRNA, messenger RNA; Sc, subcutaneous administration; siRNA, small interfering RNA.

## Appendix B

**Table A2:** Emerging lipid-lowering therapies in development stages I–III.

Emerging Therapy	Dose	Route	Drug Nature	Drug Target	Development Stage
ACP-501	13.5 mg/kg (single infusion) [57]	IV [57]	Recombinant human LCAT [57]	LCAT [57]	I [57]
ARO- ANG3	300 mg once monthly [57]	Sc [57]	siRNA [57]	ANGPTL3 [57]	I–II [57]
AZD8233	90 mg monthly [57]	PO, Sc [57]	ASO [57]	PCSK9 [57]	I–II [62]
BMS- 962476	0.3 mg/Kg every 2 weeks (assumption) [57]	Sc [57]	Adnectin [57]	PCSK9 [57]	I [57]
CSL112	6 g once weekly [57]	IV [57]	Plasma-derived ApoA-I [57,58]	ApoA-I [57,58]	II–III [57,58]
LIB003	300 mg monthly [57]	Sc [57]	Adnectin [57]	PCKS9 [57]	II–III [57]
LY3819469	Not disclosed [57]	Sc [57]	siRNA [57]	LPA [57]	I–II [57]
Obicetrapib	10 mg OD [57,63]	PO [57]	Small molecule [57]	CETP [57]	II–III [57,63]
Olpasiran	225 mg every 3 months [57]	Sc [57]	siRNA [57]	LPA [57]	II–III [57,64]
Sln360	Not disclosed [57,65]	Sc [65]	siRNA [57,58]	LPA mRNA [57,58]	I–II [57,65]
Vupanorsen	80 mg once monthly [57]	Sc [57]	ASO [57,58]	ANGPTL3 [57,58]	II [57,58]

ANGPTL3, angiopoietin-related protein 3; ApoA-I, apolipoprotein A-1; ASO, antisense oligonucleotide; CETP, cholesteryl ester transfer protein; IV, intravenous administration; LCAT, lecithin cholesterol acyltransferase; LPA, lipoprotein (a); mRNA, messenger RNA; OD, once daily; PCSK9, proprotein convertase subtilisin/kexin type 9; PO, once daily; Sc, subcutaneous administration; siRNA, small interfering RNA.

## Appendix C

**Table A3:** Additional quotes for prescribing barriers.

Participant ID	Quote
1	*The other reason might be training and development. To feel that perhaps some of the prescribers don’t feel confident, because they haven’t had the appropriate training, the appropriate CPD to underpin the knowledge, and there they may not have looked at the AAC pathway and all the lipid guidance that’s come out recently.*
2	*The most key and most impactual thing that we can do is education. Educating patients, because at the moment a lot of people don’t want to take statins because of the negative side effects that have been expressed multiple times in the media.*
	*Some people do decline it [inclisiran] when they have tried to offer it because of it being an injection form, and they don’t want to have an injection form.*
	*…with benpidoric acid. I think it’s the lack of data of there being positive results from it.*
3	*Fear of the unknown, something new [referring to novel agents].*
4	*I think a lot of patients are reluctant to have injections. They feel that it’s quite invasive. So inclisiran is something that I don’t think is too popular. But otherwise, again, with statins. I think there’s a lot of patients are worried about the side effects.*
5	*However, you know, you must stick to what you’re competent in, and I think that’s something I feel quite strongly about.*
	*So it’s still fairly, quite new to me in all honesty [novel agents], we were starting to look at the lipid pathways and what new things are on the market. So it’s only come to the practice, let’s say, probably about 3 months ago.*
	*whether or not the patient is willing to have an injection especially with the inclisiran. A lot of patients are needle phobic. They don’t want it. They don’t like the idea of having an injection.*
	*Some [patients] are really on board with it [novel therapies]. Others are not, but each patient is different. You can advise as the health care professional, but it is really down to the patient at the end of the day.*
6	*Inclisiran which is the new one. I think that’ll probably sort of once there’s more data on it, it will be routinely used. Simply because it’s 3 months then a 6 monthly dose.*
	*I think because there’s very little data at the moment. A lot of secondary care, colleagues or practices are a little bit cautious.*
	*I have discussed it with some patients who really struggle with oral and they’re not sure about having an injectable.*
	*It’s a perception with statins, the old it causes this and causes that problem… I think adherence is a big issue because of all the issues that the stigmas associated with statin treatment.*
7	*Stick to your scope of practice so the stuff you’re comfortable with.*
	*[patients] I don’t wanna increase my dose. I don’t want to get aches and pains.*
8	*the reason why I’ve been a bit more cautious with them [novel agents] is just because again, the lack of experience you know the lack of the benefit or the experience. I think as we go on, and we start prescribing it more and more. I’ll become more confident to use it. When anything’s new, your competency levels have to build up and you have to be confident of what you’re prescribing, and why you are prescribing that especially because of the cost of that medication.*
	*It’s not a cheap medication so that can play a barrier into like, you know, the surgery budget.*
	*But it’s all about shared care decisions now. So you have to come to some type of joint agreement.*
9	*There’s obviously not a lot of patients on these drugs. So from that I would say, I don’t have enough experience.*
	*I need experience of prescribing those drugs. Obviously, there’s a cost element as well.*
	*I’ve spoken to the patient he was not really interested in having an injection.*
	*I think it’s probably move. maybe because it’s a newer drug. [why patients are not keen with novel agents].*
	*I think it’s not offered because it’s new, It’s been hospital line. It’s only now sort of come into primary care. So there’s the barriers around sort of educating the clinicians around it.*
10	*I’m quite comfortable prescribing most things, so I don’t say I’m completely confident in everything, and the reason being is that mostly there’s new medications and therapies out there, and some of it’s prescribed by secondary care. So it’s quite uncommon for us to issue. so they’re always gaps in my knowledge.*
	*[patients] they read about statins and they all seem to have a negative opinion of statins and cholesterol lowering therapies. So then… when I approach the subject with them straight away, it’s can be difficult for a lot of patients. it’s almost a barrier for them.*
11	*there’s a huge amount of perceived intolerance to statins out in community.*
	*…It starts to get more and more treatments, and there is a bit push back quite right from patients and actually, I don’t wanna take all this stuff. So we need to deal with those issues as well.*
	*…it’s gonna help support us in the 2 QOF targets that we’ve got in in a GP contract. Those would be sort of drivers for us to sort of really push on and get those non-hdl levels checked and then treated.*
	*Patients have lots of variability. Some of it will just be around. poor compliance… All those sorts of stories [statin stigma] that we hear. So that sort of that influence is really strong and sometimes it can spread quite quickly in communities where we then have to really struggle to get that message sort of regressed. So people will stop to take the medication.*
12	*I suppose there’s still a barrier in the terms of its new drug. So perhaps not, all practices may be willing to prescribe or order it in.*
	*Patients sometimes aren’t keen on injections, but you have to offer the option to the patient.*
	*…couple of patients who’ve actually said no because they might have needle phobia, for example. So they’re not keen on injections. I think, also as well. I think you know at the end of the day some patients who decline statins may well decline even the injections because they don’t want to have any intervention done.*

**Table A4:** Additional quotes for prescribing enablers.

Participant ID	Quotes
1	*what can really facilitate this piece of work is having it as part of a wider team.*
	*…the more experienced you are you start to trust your decisions and believe in yourself. And I think that’s a big part of successfully being able to prescribe.*
4	*I think it’s all about the patient… Sit down, you know, ask about their concerns, and address them, and provide that reassurance that you know whatever concerns they may have we can overcome them and if they aren’t able to tolerate it, then we can always stop it.*
5	*…especially the elderly patients. You’ll find that they’ve read bad things about statins. They had statins… you know, had bad press many, many years ago and that has stuck with them. So they are, you know, really adamant that they don’t want statins, so they do like the idea of new sort of novel therapies that are coming out and they are a bit more, you know, acceptant of those medications.*
	*I think it’s all about having a conversation with a patient and explaining the benefits… For example, patients often don’t know enough about medication and they make that decision of no straight away. However, when you sit down and speak to a patient, you listen to their concerns, then you would find actually a lot of their concerns you can answer and it’s more just nerves… So it’s how you have that conversation with a patient, and I think a lot of them do understand and then go away and think about it, and they are a bit more willing to accept it.*
	*If they’ve got any sort of concerns, or, you know, worries, I always encourage my patients, you know come back to me. Let me know what your concerns or worries are. Don’t just stop taking your medication and I think they really appreciate that that they’ve got someone to talk to about those things.*
6	*…As there’s more data, more clinicians will be happy to initiate.*
	*…we’ve been doing a lot of work on educating or having kind of sessions where other clinicians and GPs are aware of the guidelines. What treatments are available and what are the latest treatments…*
	*Once you get past that first 2, 3 months with treatment patients will carry on. They won’t have a problem.*
7	*…I think, sometimes patient education and increasing the knowledge regarding the drug. Once they know what it is and why you are starting it, the potential benefits of it that can sometimes make it easier to start new medication when they’re [patients] educated, and they’ve got more information regarding it.*
8	*… We’ve received quite a bit of training now on how to prescribe [novel agents].*
	*things that make us more comfortable with these type of products is like training days, workshops.*
	*so I think like just trying to discuss it with the patient and have a follow up with the patient. So every time I start something new, I do tend to get patients to book in, like to review how they’re doing in 2 to 3 weeks and things like that if they’re still taking the medication. So it’s just follow up making sure they’re happy with still taking the medication, and then make making sure they are followed up again*
9	*They’re okay because they hear so much negative things about statins. I think they’re quite happy to go on to Inclisiran.*
	*Adherence is a massive thing and poly pharmacy. If there’s too many medications less likely to take them, so can we like optimize their care by reducing tablets.*
	*…you know, addressing your patients concerns, have they got side effects? So I think first, it’s sort of exploring the patient’s ideas, concerns, and expectations.*
	*…patient education. You know, where patients are really educated about the medicines, why they’re on it and why it starts, that you know what it reduces the risk of. You’re more likely to improve adherence.*
10	*I think we need to be flexible. So when patients say their biggest fear is getting aches and pains with statins.: if I if I feel they need them, and they’ll benefit from them. I will, you know, put my view across, and I will say to them, Oh, I can see that you’re sceptical about starting, and so I will say to them, you know, if you do run into problems, get in touch with me. I’ll tell them what the next stage is. So we can either reduce those, switch to an alternative agent or switch to an alternative medication altogether. So let them know that there are other options if they run into difficulties and if you’re approachable, and you know they know that you can be contacted should they run into difficulties they are more likely to take them.*
	*I think we just need to be clear and confident with our plan, with the patient, and share that with them, so that they know what to expect* and *telling them if they were to run into any difficulties to get in touch.*
11	*Expand it, push on it, you know, and go and develop yourself. Sit with those clinicians, whatever you need to do to expand it whats important is as non medical prescribers is we continue to work within that within that sort of scope.*
	*I think again, education is a big thing.*
	*… So we have to go out to communities to talk about some of these things.*
	*I think the important thing for us is to give… share decision making. You know. You could only have shared decision making, If you have an informed patient If you, if you have an informed patient and they decide actually, I don’t really want that. That’s fine. We have to accept that. But I think we have to give really clear guidance.*
	*it’s great to have these sort of tools [novel agents] in our armoury now.*
12	*I think education is probably the way forward, because if people understand the pathway that is around lipid management, nationally, they understand the local commissioning arrangements then its actually, it’s not too difficult to manage patients.*
	*…generally speaking, I think our job is to offer the patients the option of the novel treatments. It’s up to the patient to then decide whether they wish to take it or not. and ultimately we can’t force patients. You know. Our job is to offer them the options and then let them make an informed decision.*

**Table A5:** Additional quotes for inter-profession variability.

Participant ID	Quotes
5	*…here at the practice having pharmacists. We do have a bit more time to have that conversation with patients.*
6	*I think its [inclisiran] so new… There’s a lot of talk about it because of how effective it is But then, yeah practices and GPs are a bit cautious. So we don’t have. you know, the usual process of getting the data, you know, for it to be looked at. Safety, profile, long term effects all that. So they’re not willing to prescribe.*
9	*I don’t have experience in prescribing them and they’re newer drugs, and I don’t think the GPS have a lot of experience in prescribing them either.*
10	*…it’s a confidence issue. So if you know what you’re doing, then you’re more likely to offer alternative therapies.*

**Table A6:** Additional quotes for health literacy.

Participant ID	Quotes
1	*That shared decision making and eliciting what the patient’s concerns might be, and being able to anticipate those and deal with them effectively.*
	*I think is really important, is also making sure that there’s a level playing field. So all patients should have equal access. So whether they’re from an ethnic minority, or whether they’re from you know, a sort of deprived background.*
	*…So there might be like some practices that have, for example, and a large number of patients that are from an ethnic minority background. And for me, it’s really important that those patients get equal treatment. Somebody who’s from a more affluent area where patients might have Googled or, you know, done some research on the internet about these drugs and a more well informed, so equal access is really important as well as reducing the health inequalities. So improving health literacy would be another way of improving patient care.*
	*But explain to the patient. Why? What the benefits are? Because that will really help with the adherence, because there’s no point in escalating treatment. If we’re not dealing with the root cause of the problem which can sometimes just be like as simple as adherent, so really important that we address those issues before going into the realms of more novel agents and complex treatments and multiple therapies.*
2	*Educating patients is the most important thing.*
4	*I think, address people, you know when we get the opportunity, educating them about cholesterol and the long-term risks… and then educate them about the risks and benefits again.*
	*With satins. I think there’s a lot of patients are worried about the side effects.*
5	*I think it’s all about having a conversation with a patient and explaining the benefits. Patients often don’t know enough about medication.*
	*They [patients] have to, you know, have that understanding of why they’re on a certain type of medication. Why, they’re on lipid lowering medication, that you know it is there to prevent heart attack, stroke. Their Qrisk is usually quite high as well. So that’s the other thing that you need to kind of drum into patients is we’re not just doing it for oh, because your cholesterol is high. We’re doing it because your Qrisk is high and a lot of patients aren’t aware of that.*
6	*…they [patients] don’t know why… Why they’re having to take it. Because, you know, there’s no symptoms of high cholesterol. So you know, they’ll say, Oh, I don’t need it now.*
	*yeah, I think the biggest one is getting them to keep taking it, and patients to know why it’s so important. If they know why it’s important. Most of them do take it. It’s just that they don’t know, because they just get added in in hospital. No one really explains why they’re having to take it.*
9	*you know, when patients are really educated about the medicines, why, they’re on it and what it stops, that you know what it reduces risk of you’re more likely to improve adherence as well.*
12	*I think it’s really important to explain to them. Why are you offering it to them? Because I think a lot of patients previously were told that their cholesterol was fine. But obviously we’re now looking at cholesterol differently So I have had patients, say to me what I was told before, that my cholesterol was fine because it was below 5. Actually you have to explain to him that we now look at things like LDL and non- HDL components. Not just the total. So I think there’s a bit of patient education as well as to why we try and to use these new agents to further improve their cholesterol.*
	*I think there’s a lot of information out there on satins, for example. I mean, I think one of the important things with a lot of patients sometimes are reluctant to statins, because they’ve heard negative press about statins.*

## References

[1] Townsend N., Wilson L., Bhatnagar P., Wickramasinghe K., Rayner M., Nichols M. (2016). Cardiovascular disease in Europe: Epidemiological update 2016. Eur. Heart J..

[2] Padam P., Barton L., Wilson S., David A., Walji S., de Lorenzo F., Ray K.K., Jones B., Cegla J. (2022). Lipid lowering with inclisiran: A real-world single-centre experience. Openheart.

[3] Zhang L., Zhang S., Yu Y., Jiang H., Ge J. (2018). Efficacy and safety of rosuvastatin vs. atorvastatin in lowering LDL cholesterol A meta-analysis of trials with East Asian populations. Herz.

[4] Morrone D., Weintraub W.S., Toth P.P., Hanson M.E., Lowe R.S., Lin J., Shah A.K., Tershakovec A.M. (2012). Lipid-altering efficacy of ezetimibe plus statin and statin monotherapy and identification of factors associated with treatment response: A pooled analysis of over 21,000 subjects from 27 clinical trials. Atherosclerosis.

[5] Buckingham R. Martindale: The Complete Drug Reference.

[6] Buckingham R. Martindale: The Complete Drug Reference.

[7] Lakey W.C., Greyshock N.G., Kelley C.E., Siddiqui M.A., Ahmad U., Lokhnygina Y.V., Guyton J.R. (2016). Statin intolerance in a referral lipid clinic. J. Clin. Lipidol..

[8] Mach F., Baigent C., Catapano A.L., Koskinas K.C., Casula M., Badimon L., Chapman M.J., De Backer G.G., Delgado V., Ference B.A. (2020). 2019 ESC/EAS Guidelines for the management of dyslipidaemias: Lipid modification to reduce cardiovascular risk: The Task Force for the management of dyslipidaemias of the European Society of Cardiology (ESC) and European Atherosclerosis Society (EAS). Eur. Heart J..

[9] Ray K.K., Haq I., Bilitou A., Aguiar C., Arca M., Connolly D.L., Eriksson M., Ferrières J., Hildebrandt P., Laufs U. (2021). Evaluation of contemporary treatment of high- and very high-risk patients for the prevention of cardiovascular events in Europe-Methodology and rationale for the multinational observational SANTORINI study. Atheroscler. Plus.

[10] Anderson K.M., Wilson P.W.F., Garrison R.J., Castelli W.P. (1987). Longitudinal and secular trends in lipoprotein cholesterol measurements in a general population sample The Framingham Offspring Study. Atherosclerosis.

[11] Aygun S., Tokgozoglu L. (2022). Comparison of Current International Guidelines for the Management of Dyslipidemia. J. Clin. Med..

[12] National Institute for Health and Care Excellence (2023). Cardiovascular Disease: Risk Assessment and Reduction, Including Lipid Modification. NG238. https://www.nice.org.uk/guidance/ng238.

[13] Kotseva K., De Backer G., De Bacquer D., Rydén L., Hoes A., Grobbee D., Maggioni A., Marques-Vidal P., Jennings C., Abreu A. (2021). Primary prevention efforts are poorly developed in people at high cardiovascular risk: A report from the European Society of Cardiology EURObservational Research Programme EUROASPIRE V survey in 16 European countries. Eur. J. Prev. Cardiol..

[14] Allahyari A., Jernberg T., Hagström E., Leosdottir M., Lundman P., Ueda P. (2020). Application of the 2019 ESC/EAS dyslipidaemia guidelines to nationwide data of patients with a recent myocardial infarction: A simulation study. Eur. Heart J..

[15] Burger A.L., Pogran E., Muthspiel M., Kaufmann C.C., Jäger B., Huber K. (2022). New Treatment Targets and Innovative Lipid-Lowering Therapies in Very-High-Risk Patients with Cardiovascular Disease. Biomedicines.

[16] Tokgözoğlu L., Libby P. (2022). The dawn of a new era of targeted lipid-lowering therapies. Eur. Heart J..

[17] Agnello F., Ingala S., Laterra G., Scalia L., Barbanti M. (2024). Novel and Emerging LDL-C Lowering Strategies: A New Era of Dyslipidemia Management. J. Clin. Med..

[18] Lee G.A., Durante A., Baker E.E., Vellone E., Caggianelli G., Dellafiore F., Khan M., Khatib R. (2023). Patients’ perceptions on the facilitators and barriers using injectable therapies in dyslipidaemia: An empirical qualitative descriptive international study. J. Adv. Nurs..

[19] Lloyd-Jones D.M., Morris P.B., Ballantyne C.M., Birtcher K.K., Covington A.M., DePalma S.M., Minissian M.B., Orringer C.E., Smith S.C., Waring A.A. (2022). 2022 ACC Expert Consensus Decision Pathway on the Role of Nonstatin Therapies for LDL-Cholesterol Lowering in the Management of Atherosclerotic Cardiovascular Disease Risk: A Report of the American College of Cardiology Solution Set Oversight Committee. J. Am. Coll. Cardiol..

[20] Kakavand H., Aghakouchakzadeh M., Shahi A., Virani S.S., Dixon D.L., Van Tassell B.W., Talasaz A.H. (2020). A stepwise approach to prescribing novel lipid-lowering medications. J. Clin. Lipidol..

[21] National Institute for Health and Care Excellence (2024). Evinacumab for Treating Homozygous Familial Hypercholesterolaemia in People Aged 12 Years and over [ID2704]. GID-TA10655. https://www.nice.org.uk/guidance/indevelopment/gid-ta10655.

[22] Coleman J.J., Pontefract S.K. (2016). Adverse drug reactions. Clin. Med..

[23] Cope L.C., Abuzour A.S., Tully M.P. (2016). Nonmedical Prescribing: Where Are We Now?. Ther. Adv. Drug Saf..

[24] Community Pharmacy England (2022). Who Can Prescribe What?. https://cpe.org.uk/dispensing-and-supply/prescription-processing/receivinga-prescription/who-can-prescribe-what/.

[25] Health Education England Training for Non-Medical Prescribers. https://www.hee.nhs.uk/our-work/medicines-optimisation/training-nonmedical-prescribers.

[26] National Health Service England Independent Prescribing. https://www.england.nhs.uk/primary-care/pharmacy/pharmacy-integrationfund/independent-prescribing/.

[27] National Health Service England (2023). Changes to the GP Contract in 2023/24. https://www.england.nhs.uk/long-read/changes-to-the-gpcontract-in-2023-24/.

[28] National Health Service England (2023). Quality and Outcomes Framework Guidance for 2023/24. https://www.england.nhs.uk/wpcontent/uploads/2023/03/PRN00289-quality-and-outcomes-framework-guidance-for-2023-24.pdf.

[29] Braun V., Clarke B. (2006). Using thematic analysis in psychology. Qual. Res. Psychol..

[30] Equator Network (2021). Consolidated Criteria for Reporting Qualitative Research (COREQ): A 32-Item Checklist for Interviews and Focus Groups. https://www.equator-network.org/reporting-guidelines/coreq/.

[31] Davari M., Khorasani E., Tigabu B.M. (2018). Factors Influencing Prescribing Decisions of Physicians: A Review. Ethiop. J. Health Sci..

[32] Shafei L., Mekki L., Maklad E., Alhathal T., Ghanem R., Almalouf R., Stewart D., Nazar Z. (2023). Factors that influence patient and public adverse drug reaction reporting: A systematic review using the theoretical domains framework. Int. J. Clin. Pharm..

[33] Prosser H., Walley T. (2005). A qualitative study of GPs’ and PCO stakeholders’ views on the importance and influence of cost on prescribing. Soc. Sci. Med..

[34] Carthy P., Harvey I., Brawn R., Watkins C. (2000). A study of factors associated with cost and variation in prescribing among GPs. Fam. Pract..

[35] Dalton K., Byrne S. (2017). Role of the pharmacist in reducing healthcare costs: Current insights. Integr. Pharm. Res. Pract..

[36] National Health Service England Medicines optimisation. https://www.england.nhs.uk/medicines-2/medicines-optimisation/.

[37] Khatib R., Neely D. (2022). Summary of National Guidance for Lipid Management for Primary and Secondary Prevention of CVD. https://www.england.nhs.uk/aac/wp-content/uploads/sites/50/2020/04/lipid-managementpathway-v6.pdf.

[38] Woit C., Yuksel N., Charrois T.L. (2020). Competence and confidence with prescribing in pharmacy and medicine: A scoping review. Int. J. Pharm. Pract..

[39] Rashidian A., Omidvari A.H., Vali Y., Sturm H., Oxman A.D. (2015). Pharmaceutical policies: Effects of financial incentives for prescribers. Cochrane Database Syst. Rev..

[40] O’Donnell L.K., Ibrahim K. (2022). Polypharmacy and deprescribing: Challenging the old and embracing the new. BMC Geriatr..

[41] Iacobucci G. (2020). British GPs are more stressed and time pressured than international colleagues, survey shows. Br. Med. J..

[42] Tinelli M., Blenkinsopp A., Latter S., Smith A., Chapman S.R. (2013). Survey of patients’ experiences and perceptions of care provided by nurse and pharmacist independent prescribers in primary care. Health Expect..

[43] Stewart D.C., MacLure K., Bond C.M., Cunningham S., Diack L., George J., McCaig D.J. (2011). Pharmacist prescribing in primary care: The views of patients across Great Britain who had experienced the service. Int. J. Pharm. Pract..

[44] Gerard K., Tinelli M., Latter S., Blenkinsopp A., Smith A. (2012). Valuing the Extended Role of Prescribing Pharmacist in General Practice: Results from a Discrete Choice Experiment. Value Health.

[45] Royal Pharmaceutical Society Prescribing Competency Framework. 2016 July. https://www.rpharms.com/resources/frameworks/prescriberscompetency-framework.

[46] Graham-Clarke E., Rushton A., Noblet T., Marriott J. (2018). Facilitators and barriers to non-medical prescribing—A systematic review and thematic synthesis. PLoS ONE.

[47] Paterick T.E., Patel N., Tajik A.J., Chandrasekaran K. (2017). Improving health outcomes through patient education and partnerships with patients. Bayl. Univ. Med. Cent. Proc..

[48] Wood F.A., Howard J.P., Finegold J.A., Nowbar A.N., Thompson D.M., Arnold A.D., Rajkumar C.A., Connolly S., Cegla J., Stride C. (2020). N-of-1 Trial of a Statin, Placebo, or No Treatment to Assess Side Effects. N. Engl. J. Med..

[49] Tarn D.M., Barrientos M., Pletcher M.J., Cox K., Turner J., Fernandez A., Schwartz J.B. (2021). Perceptions of Patients with Primary Nonadherence to Statin Medications. J. Am. Board Fam. Med..

[50] National Health Serice England Deprivation. https://www.england.nhs.uk/about/equality/equality-hub/national-healthcare-inequalitiesimprovement-programme/what-are-healthcare-inequalities/deprivation/.

[51] National Health Service England NHS Workforce Race Equality Standard. https://www.england.nhs.uk/about/equality/equality-hub/workforceequality-data-standards/equality-standard/.

[52] Green J., Thorogood N. (2018). Qualitative Methods for Health Research.

[53] Corbin J., Strauss A. (2014). Basics of Qualitative Research: Techniques and Procedures for Developing Grounded Theory.

[54] OpenPrescribing. https://openprescribing.net/analyse/#org=regional_team&numIds=0212000AMAA&denom=nothing&selectedTab=summary.

[55] OpenPrescribing. https://openprescribing.net/analyse/#org=regional_team&numIds=0212000AK&denom=nothing&selectedTab=summary.

[56] OpenPrescribing. https://openprescribing.net/analyse/#org=regional_team&numIds=0212000ALAA&denom=nothing&selectedTab=summary.

[57] Brandts J., Ray K.K. (2023). Novel and future lipid-modulating therapies for the prevention of cardiovascular disease. Nat. Rev. Cardiol..

[58] Kim K., Ginsberg H.N., Choi S.H. (2022). New, Novel Lipid-Lowering Agents for Reducing Cardiovascular Risk: Beyond Statins. Diabetes Metab. J..

[59] National Library of Medicine (2020). Omega 3 and Ischemic Stroke; Fish Oil as an Option (OmegaStroke). https://clinicaltrials.gov/study/NCT04386525?intr=Omega-3%20fatty%20acids%20(DHA%20and%20EPA)&aggFilters=phase:4&rank=7.

[60] National Library of Medicine (2022). A Study of Olezarsen (ISIS 678354) in Participants with Hypertriglyceridemia and Atherosclerotic Cardiovascular Disease, or with Severe Hypertriglyceridemia. https://clinicaltrials.gov/study/NCT05610280?intr=olezarsen&rank=57.

[61] National Library of Medicine (2023). A Phase III Confirmatory Study of K-877 (Pemafibrate) in Patients with Hypercholesterolemia and Statin Intolerance. https://clinicaltrials.gov/study/NCT05923281?intr=pemafibrate&aggFilters=phase:3&rank=37.

[62] National Library of Medicine (2020). A Study of AZD8233 in Participants with Dyslipidemia. https://clinicaltrials.gov/study/NCT04641299?intr=AZD8233&aggFilters=phase:2&rank=17.

[63] National Library of Medicine (2022). Cardiovascular Outcome Study to Evaluate the Effect of Obicetrapib in Patients with Cardiovascular Disease (PREVAIL). https://clinicaltrials.gov/study/NCT05202509?intr=Obicetrapib&aggFilters=phase:3&rank=37.

[64] National Library of Medicine (2022). Olpasiran Trials of Cardiovascular Events and Lipoprotein(a) Reduction (OCEAN(a))—Outcomes Trial. https://clinicaltrials.gov/study/NCT05581303?intr=Olpasiran&aggFilters=phase:3&rank=17.

[65] National Library of Medicine (2023). Evaluate SLN360 in Participants with Elevated Lipoprotein(a) at High Risk of Atherosclerotic Cardiovascular Disease Events. https://clinicaltrials.gov/study/NCT05537571?intr=Sln360&rank=17.

