# Supplementary material for: Exploring the Perceptions and Behaviours of UK Prescribers Concerning Novel Lipid-Lowering Agent Prescriptions: A Qualitative Study
Source: Pharmacy (Basel). 2024 Jul 3;12(4):104. doi: 10.3390/pharmacy12040104 (PMC11270282; doi:10.3390/pharmacy12040104)
Supplement: Supplementary file 1 [file pharmacy-12-00104-s001.zip › pharmacy-2979342-supplementary.pdf]

Interview topic guide

1. What is your professional background?
    - How many years have you been in practice?
    - How many years have you been prescribing?
    - What does your role entail?
    - Do you have a specialty?
    - Why did you choose this particular specialty?
2. Could you please discuss how confident you feel in prescribing and why?
    - On average how often do you prescribe lipid lowering therapy?
3. How competent do you feel in prescribing?
    - Can you please describe your training in prescribing?
4. Please can you share your knowledge and experience regarding novel lipid therapies?

Prompts—bempedoic acid or inclisiran?

- How often have you prescribed novel lipid therapies like bempedoic acid and why? If this is not the case, can you explain why you may have not prescribed either drug?
- Are these drugs present on your local formulary?
- Can you please describe a patients typical therapy journey before novel lipid therapies are considered i.e bempedoic acid or inclsirian is considered?

5. In your opinion, are there any difficulties from a prescribers' viewpoint that can decrease the rate of prescribing novel lipid therapies, e.g., bempedoic acid or inclisiran?

Prompts: environmental, personal, or organizational.

- What can be done to overcome these barriers from a prescribers' point of view?

6. In your opinion, are there any difficulties from a patients' point of view, which can lead to refusing treatment of these drugs?
    - What can be done to overcome these difficulties for patients to optimise their therapy?
7. Can you please tell me any commonalities you have noticed amongst patients who require novel lipid therapies, like bempedoic acid or inclisiran?
    - Are patients in a specific age bracket affected more than others? If so, why do you think this is the case?
    - In your experience, can you describe any common modifiable or unmodifiable factors which can lead to prescribing these newer agents?
    - What informs your decision making in prescribing these novel agents for example are certain blood markers commonly raised above a certain range?
8. Can you please tell me what more can be done from HCPs to help target cholesterol levels before they reach a stage that becomes difficult to treat?
    - In your opinion, what can be done more from a patients point of view to not reach this stage?

Closing statement

Thank you for your time, your responses have been extremely valuable. Before we finish this interview, is there anything else you would like to discuss? Once again, thank you for your time.
